# Translation, Cultural Adaptation and Validation of the Basic Psychological Needs Satisfaction in Active Commuting to and From School (BPNS-ACS) Scale in Polish Students

**DOI:** 10.34763/jmotherandchild.2021.2503SI.d-21-00030

**Published:** 2022-02-09

**Authors:** Anna Dzielska, Agnieszka Michalska, Dorota Kleszczewska, Dorothea M.I. Schönbach, Adilson Marques, Miguel Peralta, Yolanda Demetriou

**Affiliations:** 1 Department of Child and Adolescent Health, Institute of Mother and Child, Warsaw, Poland; 2 Department of Biomedical Foundations of Development and Sexology, Faculty of Education, University of Warsaw, Warsaw, Poland; 3 Institute of Mother and Child Foundation, Warsaw, Poland; 4 Department of Sport and Health Sciences, Technical University of Munich, Munich, Germany; 5 ISAMB, Faculty of Medicine, University of Lisbon, Lisbon, Portugal; 6 CIPER, Faculty of Human Kinetics, University of Lisbon, Lisbon, Portugal

**Keywords:** BPNB-ACS, Validation, Self-determination theory, Active commuting to school, Physical activity, Adolescents

## Abstract

**Background:**

To promote active commuting to and from school, it is pertinent to understand the motivational factors that influence the choice of this form of transportation.

**Objective:**

Translation, cultural adaptation and analysis of the factor structure as well as psychometric properties of the Basic Psychological Needs and Satisfaction in Active Commuting to and from School (BPNS-ACS) scale among Polish students and examination of the distribution of the scale scores according to gender, mode of commuting to and from school and the frequency of using bicycle for this purpose.

**Material and methods:**

Data from 475 Polish students aged 11–18, including 53.9% of girls were analysed. The Confirmatory Factor Analysis of the Polish version of the BPNS-ACS, U Mann-Whitney and Kruskal Wallis H tests were performed.

**Results:**

The BPNS-ACS consists of 12 items forming three dimensions: autonomy, competence and relatedness need satisfaction. The scale has acceptable psychometric properties: χ2(51)=195.424 (p ˂ 0.001); χ2/df=3.832; CFI=0.944, TLI=0.927, RMSEA=0.077 (90%CI 0.066-0.089), AIC=249.424, BIC=361.833, GFI=0.937, AGFI=0.904. BPNC-ACS scores on factors corresponding to the three basic psychological needs differ based on gender, mode of commuting to and from school and the frequency of cycling to or from school.

**Conclusions:**

Further exploration of the function of basic psychological needs in active commuting to and from school among Polish adolescents may be conducted using an adapted version of the BPNS-ACS scale.

## Introduction

Over the last few years, there has been an alarming decline in physical activity among adolescents described as insufficient for maintaining health [1]. Physical activity is an essential component of a physical and mental health of children and adolescents. As evidenced by the results of a survey of 171,000 adolescents from 37 countries, adolescents who have active and healthy lifestyles are less likely to experience psychosomatic disorders [2]. Further, physical activity is an important component of efforts to maintain psychosocial well-being among the adolescents [3]. It has also been proven that physical activity at an adequate level is a key component of prevention, helping to reduce the incidence of non-communicable diseases throughout life [4].

The level of physical activity is mainly determined by the individual's motivation, the level of satisfaction of his/her basic psychological needs (BPN), causing commitment [5]. In the light of the Self-Determination Theory (SDT) by Deci and Ryan [6], there is an opportunity for an in-depth analysis of motivation and personal development components in different contexts of an individual's daily functioning. One of the core concepts in the SDT is the concept of BPN, defined as innate, universal and indispensable to human psychological well-being [7]. BPNs are natural stimuli that influence an individual's type of motivation regulation. Within the BPN theory, three components are distinguished: 1) autonomy, 2) competence and 3) relatedness. The satisfaction of BPN leads to an increase in sense of autonomy, competence and relatedness, which in turn affects a higher level of autonomous motivation, self-control and activity understood as taking action [8]. Therewith, their satisfaction also provides the basis for maintaining mental health. This is of particular importance in the context of school health, which consists of the promotion of active commuting to school (ACS) among the younger generation.

Motivation plays an important role among students when deciding to actively commute to and from school. Active commuting, which involves primarily walking and cycling, is one component of an active lifestyle that can easily be incorporated into daily routines among students [9, 10, 11]. Each of these modes has been identified as a potential way to increase physical activity levels, providing an alternative to more traditional ways of being physically active [12, 13]. Active modes of commuting to and from school were identified as a potential opportunity to increase physical activity in students [14]. In addition, active commuting to and from school as cycling or walking may contribute to meeting physical activity recommendations for the younger generation and thus may increase physical activity in students at the population level [15].

BPN satisfaction, self-determined motivation and engagement occur among students who make the decision to actively commute to school [16]. In order to be able to consciously promote active commuting among the younger generation, it is useful to understand the motivational factors that facilitate or hinder their choices [17].

The main goal of the study was the translation, cultural adaptation and analysis of the factor structure and psychometric properties of the BPNS-ACS scale in a sample of Polish students. The distribution of BPNS-ACS scale scores by gender and measures related to active commuting to and from school were also examined. The evaluation of the BPNS-ACS scale in Polish students also provided an opportunity to discuss the practical use of this instrument in planning intervention activities.

## Material and methods

### Participants

The study was conducted in Poland in June 2021. Overall, 475 students participated, including 53.9% of girls, from 13 primary and secondary schools. The age of students ranged from 11 to 18 years (M_age=_14,2, SD_age_=1,9). Students filled in the questionnaire at school under the supervision of a researcher (AD). Parents and students aged ≥ 13 years were informed about the purpose of the study and its circumstances in advance and gave their informed consent to participate in accordance with the Helsinki Declaration. It was possible to opt out of the study at any time during its course without giving any reason and without any consequences. The procedure and the tools used in the research project were approved by the Bioethics Committee of the Institute of Mother and Child in Warsaw, Poland (No. 51/2021 from 24.06.2021).

### Translation and cultural adaptation procedure

The cultural adaptation was prepared according to the guidelines for linguistic adaptation of scales and questionnaires in epidemiological studies [18] using several stages, including theoretical construct analysis and source analysis, straightforward translation made by two independent translators, unifying the target working version, back-translation, comparison of reverse translations with the original version, unification of the target language version, quantitative and qualitative study, final version, psychometric analysis, validation and dissemination of the final version.

### Instruments

The questionnaire consisted of questions about health behaviors of students and included, among others, questions constituting the BPNS scale and active commuting to and from school.

The Polish version of the BPNS-ACS scale was created on the basis the Spanish instrument [10], which was developed by adapting the Spanish version of the Basic Psychological Needs in Exercise Scale (BPN-ES) to the context of ACS [19]. The questionnaire consists of 12 items grouped according to the three components of BPNs, i.e autonomy [autonomy need satisfaction (ANS)], competence [competence need satisfaction (CNS)], and relatedness [relatedness need satisfaction (RNS)]. Questions pertaining to autonomy are related to student’s self-determination, e.g. ‘I feel that my usual mode of commuting to and from school fits well with what I want’. The component of competence refers to both skills and physical and mental resources of students, e.g. ‘I feel able to walk or cycle to and from school’. The last component called relatedness represents the social aspect of the activity, e.g. ‘I feel very comfortable with who accompanies me to school’. The questionnaire used a five-point Likert scale ranging from 1 (totally disagree) to 5 (totally agree). The summary index of three factors (scales) ranged from 4 to 20 points. The full wording of all items is included in Table 1 as supplementary material.

**Table 1:** English language version of the BPNS-ACS scale with a translation into Polish Tabela 1. Angielska wersja językowa skali BPNS-ACS wraz z tłumaczeniem na język polski

	BPNS-ACS – English version	BPNS-ACS –Polish version
	**Autonomy Need Satisfaction**	

ITEM 1	I feel that my usual mode of commuting to and from school fits well with what I want	Mam poczucie, że sposób, w jaki zwykle pokonuję drogę do i ze szkoły, jest zgodny z tym czego chcę
ITEM 4	I feel that the mode of commuting to and from school coincides with how I want to travel	Mam poczucie, że sposób, w jaki pokonuję drogę do i ze szkoły odpowiada temu, jak chcę podróżować
ITEM 7	I feel that the mode of commuting to and from school is what I like	Mam poczucie, że lubię sposób, w jaki pokonuję drogę do i ze szkoły
ITEM 10	I feel that I can choose how to commute to and from school	Mam poczucie, że mogę wybrać sposób w jaki pokonuję drogę do szkoły i ze szkoły

	**Competence Need Satisfaction**	

ITEM 2	I feel able to walk or cycle to and from school	Mam poczucie, że jestem w stanie chodzić pieszo lub jeździć rowerem do i ze szkoły
ITEM 5	I feel that I have the necessary skills to walk or cycle to and from school	Mam poczucie, że mam niezbędne umiejętności, aby chodzić pieszo lub jeździć rowerem do i ze szkoły
ITEM 8	I feel skilled to walk or cycle to and from school	Czuje się na siłach żeby chodzić pieszo lub jeździć rowerem do i ze szkoły
ITEM 11	I feel competent to walk or cycle to and from school	Czuję, że mam umiejętności, żeby chodzić pieszo lub jeździć rowerem do i ze szkoły

	**Relatedness Need Satisfaction**	

ITEM 3	I feel extremely comfortable when someone accompanies me to school	Czuję się bardzo komfortowo, gdy ktoś towarzyszy mi w drodze do szkoły
ITEM 6	I friendly interact with who accompanies me to school	Odnoszę się w przyjazny sposób do osoby, która towarzyszy mi w drodze do szkoły
ITEM 9	I feel that I can openly communicate with who accompanies me to school	Mam poczucie, że mogę otwarcie rozmawiać z osobą, która towarzyszy mi w drodze do szkoły
ITEM 12	I feel very comfortable with who accompanies me to school	Czuje się swobodnie z osobą, która towarzyszy mi w drodze do szkoły

**Question**: English: How do you feel about the mode you typically choose to get TO and FROM school? Choose a single answer in each row. Polish: Co sądzisz o swoim typowym sposobie pokonywania drogi DO i ZE szkoły? Zaznacz znakiem X jedną odpowiedź w każdym wierszu). **Answer categories**: English: 1 Strongly DISAGREE; 2; 3; 4; 5 Strongly AGREE Polish: 1 Zdecydowanie się NIE zgadzam; 2; 3; 4; 5 Zdecydowanie się zgadzam

### Measures related to active commuting to school

Students were asked for their mode used to commute to and from school (i.e. by foot, bike, car, motorbike/scooter/ or bus/train/tram). The first two categories were classified as active modes of commuting, and the remaining categories were classified as passive modes of commuting. About 48.4% and 51.6% percent of students, respectively, fell into the aforementioned categories.

Students were also asked to report the number of days, from 0 to 5, on which they usually cycle to school. This variable was analysed based on three categories: do not cycle to school at all (0 days), cycle to school sometimes (1–2 days), cycle to school frequently (3 days or more). The proportion of students in the following groups was 60.4%, 10.1% and 24.8%, respectively.

### Data analysis

Two scale attenuation effects were analysed: floor effects (percentage of the lowest modality) and ceiling effects (percentage of the highest modality). The effects of maximum 15% were considered as acceptable [20]. The skew and the kurtosis values for each item were used to evaluate normality for each item. The component reliability was analysed representing z-values (Z) for testing whether the univariate distribution for an item departs significantly from normality with respect to skew and kurtosis. A value of Z_skew_ between -2 and +2 indicated a normality of the distribution [21], and a value > 3 indicated severe non-normality [22]. The value of Z_kurtosis_ between -2 and +2 also indicated normality [21], but severe non-normality was confirmed by the result of ≥ 7 [22]. An accepted value for Z_kurtosis_ was ≥ 7 [23].The factor structure and the psychometric properties of the Polish version of the scale were analysed using a confirmatory factor analysis (CFA). The purpose of using a CFA was to confirm that the number of factors and loadings of observed variables were consistent with theoretical assumptions [10]. Parameter values were estimated using the maximum likelihood method.

Moreover, due to the lack of multivariate normality, a bootstrapping procedure was implemented and set on 5,000 replication samples with 95% confidence intervals (CIs) for all statistical parameters. The following model fit indices were analysed: chi-square test (χ 2/df); comparative fit index (CFI), goodness-of-fit index (GFI), adjusted goodness-of-fit index (AGFI), normed fit index (NFI), Tucker–Lewis index (TLI) and root mean square error of approximation (RMSEA).

The reliability and validity of the scale were assessed by calculating component reliability (CR), average variance extracted (AVE), and the Cronbach’s α coefficient. Values of AGFI, GFI, CFI, TLI and NFI ≥ 0.90 indicated good and adequate adjustment of the model to data [23]. A value of χ 2/df < 2 also suggested a good fit of the model to data. A value of RMSEA < 0.08 was also interpreted as a sufficient fit to data [22, 23]. Effect sizes were evaluated with Cohen’s d. Effects with d=0.20 to 0.50 were interpreted as small, effects with d=0.50 to 0.80 as medium and effects with d > 0.8 as large [24]. An accepted value for Cronbachs-α was ≥ 7 and for AVE > 0.5 [23].

To test differences in scores of the analysed scale and its factors by gender and the measures related to the fact of commuting to school and frequency of using bicycle for this purpose, Mann-Whitney U and Kruskal-Wallis H non-parametric tests were used [25]. Pairwise comparisons were performed using Dunn's (1964) procedure with a Bonferroni correction for multiple comparisons [26].

Statistical analyses were performed using AMOS 25.0 and IBM SPSS Statistics for Windows, Version 26.0. Armonk, NY: IBM Corp.

## Results

Table 1 shows the basic descriptive statistics for the analysed items as well as a floor and ceiling effects. The distribution of analysed items was observed to differ significantly from a normal distribution. The ceiling effect was high on all studied items, and the floor effect was particularly visible in items 2, 4, 5, 6 and 7.

**Table 1:** Descriptive statistics, floor and ceiling effects for individual items (N=475)

	M	SD	Skew	Z _Skew_	Kurtosis	Z_Kurtosis_	Floor Effect (%)	Ceiling Effect (%)
**Item 1**	4.21	1.387	-1.555	-13.837	0.855	3.805	12.6	68.6
**Item 2**	3.73	1.629	-.978	-8.703	-0.816	-3.631	21.5	54.5
**Item 3**	4.05	1.446	-1.455	-12.944	0.590	2.625	13.7	62.3
**Item 4**	3.86	1.603	-.807	-7.184	-1.045	-4.648	18.1	60.6
**Item 5**	3.87	1.632	-1.186	-10.557	-0.321	-1.428	20.6	61.7
**Item 6**	4.01	1.551	-1.786	-15.893	1.837	8.174	17.3	65.7
**Item 7**	3.87	1.630	-1.238	-11.013	-0.011	-0.049	20.4	61.7
**Item 8**	4.13	1.465	-1.398	-12.442	0.292	1.301	14.7	68.0
**Item 9**	4.17	1.390	-1.116	-9.927	-0.396	-1.76	12.2	66.7
**Item 10**	4.32	1.250	-.933	-8.300	-0.867	-3.859	8.6	70.5
**Item 11**	3.96	1.515	-.975	-8.673	-0.819	-3.643	15.8	60.6
**Item 12**	4.21	1.350	-1.522	-13.542	0.844	3.753	10.9	67.7

### Reliability and validity

Descriptive statistics of the three factors of the BPNS-ACS scale are shown in Table 2. Analysis of reliability showed that two of three factors have good component reliability (0.715 for CNS and 0.775 for RNS), but the value of CR for ANS was not satisfactory (0.568). The AVE values indicated good construct validity of all factors ranging from 0.439 to 0.621. The values of internal consistency indicated good reliability for all three factors (Cronbach’s-α=0.744 for ANS, Cronbach’s-α=0.859 for CNS, and Cronbach’s-α=0.865 for RNS).

**Table 2:** Descriptive statistics of the three factors of the BPNS-ACS scale

	Autonomy Need Satisfaction	Competence Need Satisfaction	Relatedness Need Satisfaction
M (SD)	15.84 (4.57)	15.88 (5.27)	16.66 (4.65)
Skew	-1.003	-1.056	-1.355
Kurtosis	0.137	-0.197	0.840
CR	0.568	0.715	0.775
AVE	0.439	0.610	0.621
Cronbach’s-α	0.744	0.859	0.865

### Model fit assessment

**Figure 1:**
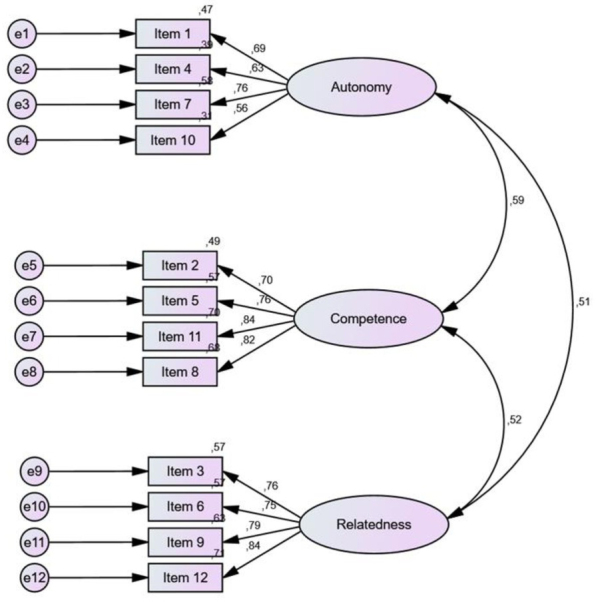
Three-factor BPNS-ACS model

The χ2 test was statistically significant (χ^2^(51)=195.424 (p ˂ 0.001); χ^2^/df=3.832), but the value of χ^2^/df was equal to 3.83, which indicates good adjustment of the model. Moreover, the absolute fit and incremental fit parameters of the model indicated a good fit (CFI=0.944, TLI=0.927, RMSEA=0.077 (90%CI 0.066-0.089), AIC=249.424, BIC=361.833, GFI=0.937, AGFI=0.904). The values of standardised regression coefficients ranged from 0.56 to 0.84 (Figure 1).

### BNPN-ACS distribution by gender and active commuting to school

Analysis of the distributions of the scores in the subsequent BPNS-ACS factors showed that the scores for males and females were not similar only in the case of ANS. The scores for boys (mean rank=253.52) were higher than for girls (mean rank=224.72), U=24632.500, z=2.353, p=0.019. No statistically significant differences related to gender were found in case of the CNS or RNS scores.

When considering active commuting to and from school, statistically significant differences in scale scores between those who do not actively commute (mean rank=197.11) and those who actively commute to and from school (mean rank=276.39) were observed for the CNS factor, U=37.590.500, z=6.687, p=0.000. No statistically significant differences were found related to ACS in the ANS or RNS scores.

A Kruskal-Wallis H test was calculated to determine if there were differences in scores of ANS, CNS, and RNC between three groups of students classified into different groups according to their frequency of cycling to/from school. Mean rank of the ANS and ANS scores were statistically significantly different between groups dividing by the frequency of cycling, χ2(2)=7.927, p=0.019 and χ2(2)=13.121, p=0.001, respectively. Distributions of RNS scores were similar for all groups and the mean rank of its scores was not statistically significantly different between groups, χ2(2)=0.680, p=0.710. The post hoc analysis revealed statistically significant differences in ANS scores between students who do not cycle (215.07) and those who cycle frequently (257.83), (p =0.005), but not between any other group combinations. Similar results were observed in differences of CNS. Statistically significant results were observed only between the groups of students who did not cycle to or form school (213.13) and cycled frequently (261.85), (p=0.000).

## Discussion

Assessing students’ BPNs satisfaction may be an important component of efforts aimed at understanding students’ behaviour regarding ACS. This scope should play a significant role around diagnosis of primordial factors associated with students' autonomous choice of active commuting. Active commuting including walking and cycling to and from school are important modes of engagement in daily physical activity [27]. There is ample evidence that walking and cycling are associated with positive health outcomes, which include mental well-being, healthy body weight, reduced risk of chronic disease, and better overall health [28, 11, 29]. Motivation, a key factor supporting engagement in physical activity, simultaneously implies positive health outcomes [30]. This is particularly relevant to the implementation of activities aimed at increasing awareness and potential involvement of students in active modes of commuting to and from school as part of health promotion [31].

The inclusion of assessing the satisfaction of BPNs in the context of active modes of commuting to school among students is a relatively new research approach, and as a result, the number of validated tools for assessing the satisfaction of BPNs in this context is small. In 2012, the validation of a similar scale, being in the context of physical activity, was carried out by researchers whose aim was to determine the level of activity around physical exercise among a population of Spanish adults, considering the satisfaction of their BPNs. The results obtained after the analysis of psychometric properties confirmed the adequacy of the tool [32]. With reference to the current study on the validation of the BPNS-ACS scale and its psychometric properties among 675 Spanish students (318 boys and 357 girls) aged between 10 and 18 years, the results clearly confirmed the validity of the three-factor CFA model and the predictive accuracy of the present tool. Furthermore, no specification errors were found for this factor model. The validation of the BPN-ACS scale in Spanish students in 2020 proved that it is a tool providing temporal stability, measurement invariance for gender and age, and internal consistency [10]. Our analyses of Polish students led to similar conclusions, and the results suggest that the Polish version of the scale can be used as a reliable measure to assess each of the three BPNs, i.e. autonomy, competence and relatedness, in the context of ACS.

It is worth noting that the BPNS-ACS contains many similar-sounding questions. These are questions that form further dimensions of the scale: autonomy need satisfaction, competence need satisfaction and relatedness need satisfaction. This may not be distinguishable by the respondents and may influence the answer choices to become random. To avoid overlaps in the items, attention should be paid to the proper ordering of questions in the questionnaire, consistent with the order proposed in the appendix of this work, so that questions included in successive dimensions of the scale do not occur immediately after each other, but along to questions from other dimensions.

Studies based on the SDT confirm that students who evaluate their BPNs of autonomy, competence and relatedness as satisfied are more committed and motivated to engage in physical activity [33]. The development of a comprehensive conceptual framework for intervention programs that integrates work on BPNs could support the development of appropriate strategies to promote students’ health, including modelling modes of active commuting in students. Future research efforts should therefore be directed towards analyses concerning the influence of individual elements of the students’ life environment, by what the satisfaction of these three key BPNs has an impact on the formation of motivation to act [3]. This is particularly important from the perspective of projecting the actions taken by educational and health professionals and enabling them to practically implement strategies based on the motivation of students by, among other things, providing a conducive school environment for the promotion of physical activity [34, 27]. The thesis on the effectiveness of interventions considering working on needs in relation to improving physical activity is supported by research findings [35]. In addition to physical education classes, it is also important to ensure that components of the school environment are adequately prepared to meet the needs of students in terms of active commuting as an additional opportunity of physical activity. Especially since adolescence is an important time for structured actions oriented towards the promotion of physical activity as a long-term investment for health [36]. When analysing international ACS indicators, researchers describe them as still insufficient, especially when combined with high rates of passive modes of commuting to/from school, including the widespread use of private vehicles for this purpose [37, 38].

An interesting area for further research using the BPNS-ACS scale seems to be determining what BPNs are most important in the context of ACS. Our findings suggest that the distribution of individual scale scores corresponding to factors in the BPNs-ACS scale differ by gender as well as with respect to active commuting to and from school. Significantly higher scores on the autonomy scale were recorded for boys. In addition, active commuters had higher scores on the competence scale than passive commuters. Furthermore, the frequency of cycling to school was important. Students who cycle to school obtain higher scores on the scale of autonomy and competence compared with their peers who do not actively commute.

In conclusion, research exploring the interrelationship between BPNs and ACS is important for new designs and implementations of strategies to support the promotion of active commuting to and from school. A further exploration of this issue can be conducted in Polish students using the version of the BPNS-ACS scale adapted in this study.

## Key points

· The Polish version of the BNPS-ACS scale is an important instrument for measuring basic psychological needs in terms of commuting to and from school by students.
· Further exploratory research using the BPNS-ACS scale is needed to determine which basic psychological need, and in what context, is most important for choosing active modes of commuting to and from school.
· The context of basic psychological needs should be an important component of intervention programs towards increasing adolescents' use of active commuting modes.
